# Association of Mobile-Enhanced Remote Patient Monitoring with Blood Pressure Control in Hypertensive Patients with Comorbidities: A Multicenter Pre–Post Evaluation

**DOI:** 10.3390/diagnostics16020244

**Published:** 2026-01-12

**Authors:** Ashfaq Ullah, Irfan Ahmad, Wei Deng

**Affiliations:** 1 Department of General Practice, Second Affiliated Hospital of Chongqing Medical University, Chongqing 400010, China; ashfaq@stu.cqmu.edu.cn; 2 Department of Rehabilitation Medicine, Second Affiliated Hospital of Chongqing Medical University, Chongqing 400010, China; 27irfanpeace@gmail.com

**Keywords:** mobile technology (MT), hypertension (HT), coexistence of multiple diseases (CMD), health management (HM), remote patient monitoring (RPM)

## Abstract

**Background and Objectives:** Hypertension affects more than 27% of adults in China, and despite ongoing public health efforts, substantial gaps remain in awareness, treatment, and blood pressure control, particularly among older adults and patients with multiple comorbidities. Conventional clinic-based care often provides limited opportunity for frequent monitoring and timely treatment adjustment, which may contribute to persistent poor control in routine practice. The objective of this study was to evaluate changes in blood pressure control and related clinical indicators during implementation of a mobile-enhanced remote patient monitoring (RPM)–supported care model among hypertensive patients with comorbidities, including patterns of medication adjustment, adherence, and selected cardiometabolic parameters. **Methods:** We conducted a multicenter, pre–post evaluation of a mobile-enhanced remote patient monitoring (RPM) program among 6874 adults with hypertension managed at six hospitals in Chongqing, China. Participants received usual care during the pre-RPM phase (April–September 2024; clinic blood pressure measured using an Omron HEM-7136 device), followed by an RPM-supported phase (October 2024–March 2025; home blood pressure measured twice daily using connected A666G monitors with automated transmission via WeChat, medication reminders, and clinician follow-up). Given the use of different devices and measurement settings, blood pressure comparisons may be influenced by device- and setting-related measurement differences. Monthly blood pressure averages were calculated from all available readings. Subgroup analyses explored patterns by sex, age, baseline BP category, and comorbidity status. **Results:** The cohort was 48.9% male with a mean age of 66.9 ± 13.7 years. During the RPM-supported care period, the proportion meeting the study’s blood pressure control threshold increased from 62.4% (pre-RPM) to 90.1%. Mean systolic blood pressure decreased from 140 mmHg at baseline to 116–118 mmHg at 6 months during the more frequent monitoring and active treatment adjustment period supported by RPM (*p* < 0.001), alongside modest reductions in fasting blood glucose and total cholesterol. These achieved SBP levels are below commonly recommended office targets for many older adults (typically <140 mmHg for ages 65–79, with individualized lower targets only if well tolerated; and less stringent targets for adults ≥80 years) and therefore warrant cautious interpretation and safety contextualization. Medication adherence improved, and antihypertensive regimen intensity increased during follow-up, suggesting that more frequent monitoring and active treatment adjustment contributed to the early blood pressure decline. Subgroup patterns were broadly similar across age and baseline BP categories; observed differences by sex and comorbidity groups were exploratory. **Conclusions:** In this large multicenter pre–post study, implementation of an RPM-supported hypertension care model was associated with substantial improvements in blood pressure control and concurrent intensification of guideline-concordant therapy. Given the absence of a concurrent control group, clinic-to-home measurement differences, and concurrent medication changes, findings should be interpreted as associations observed during an intensified monitoring and treatment period rather than definitive causal effects of RPM technology alone. Pragmatic randomized evaluations with standardized measurement protocols, longer follow-up, and cost-effectiveness analyses are warranted.

## 1. Introduction

Hypertension is a primary risk factor for cardiovascular diseases, stroke, and renal failure. It is a major public health concern, affecting 1.28 billion people, with control rates below 20% globally [1]. In China, the prevalence of hypertension among adults is more than 27%, with very low control rates [2,3]. Hypertension management is frequently exacerbated by the presence of multiple chronic diseases, such as diabetes, obesity, and dyslipidemia, which require continuous control [4,5]. Machine learning algorithms using body mass index (BMI) and comorbidity data have predicted hypertension progression with 82% accuracy, highlighting the necessity for risk stratification in complex patients [6]. In addition, integrated care models can effectively control comorbidities and minimize the burden on hospitals [7,8]. Conventional healthcare systems often fail to provide real-time monitoring, optimal blood pressure management, and risk reduction [9]. Remote patient monitoring (RPM) enhanced by wearable technologies is an innovative technique for chronic disease management. It increases patient engagement and medication adherence [10]. Randomized and controlled studies indicate that home BP telemonitoring, especially when paired with structured clinician support or medication titration, improves BP control compared with usual care. However, average effect sizes are typically modest and heterogeneous across implementations. Key examples include TASMINH4 (self-monitoring with/without telemonitoring to guide titration) and pharmacist-supported telemonitoring models such as HyperLink, both demonstrating improved BP control versus usual care [11,12,13]. However, less is known about how RPM performs in routine, multicenter Chinese settings using widely adopted platforms (e.g., WeChat), particularly among older patients with comorbidities and when outcomes include not only BP but also medication adjustments, adherence patterns, and cardiometabolic markers. By enabling frequent home BP measurement with automated transmission and by supporting structured clinician review and timely follow-up, RPM can facilitate earlier treatment adjustment and reinforce self-management. It can also detect early fluctuations and facilitate treatment modification and clinical decision-making to prevent serious complications [14,15]. An elevated BMI, especially in diabetic patients, enhanced the risk of hypertension by 35% emphasizing the need for AI-driven weight management techniques [16,17].

RPM can help in chronic disease management by assisting in early diagnosis, treatment, prevention, and risk factor identification [18]. These wearable devices, mobile applications, and electronic health records also slow down the progression of chronic diseases and improve health outcomes [19,20]. This integration empowers patients and healthcare providers to enhance care delivery [21].

Chongqing is a large municipality with pronounced variation in healthcare access across dense urban districts and outlying peri-urban and rural areas. Recent analyses have highlighted inequities in local health resource allocation and spatial accessibility, which can translate into uneven continuity of chronic disease follow-up [22,23]. In this context—within China’s three-tier system in which community health centers deliver a substantial share of chronic disease management—RPM is particularly informative because it can extend follow-up capacity beyond face-to-face visits, support standardized monitoring across sites, and reduce the burden of travel and clinic crowding while maintaining clinician oversight through common digital platforms such as WeChat [24,25].

Wearable devices collect and transmit data to the monitoring platform, providing continuous monitoring and fast tracking. The monitoring platform sends regular reminders for BP measurement and to take antihypertensive medication [26,27]. RPM increases patients’ health literacy by providing access to information and real-time feedback about their health status, which is essential for chronic disease management [28,29,30,31,32]. Community health centers receive patients’ data at the monitoring platform, and medical staff immediately respond to abnormal BP recordings, thereby reducing the major cardiovascular accidents [33,34]. Furthermore, RPM uses AI-assisted care coordination, reducing financial burden and hospital readmissions by 28% among hypertensive patients with comorbidities [35,36,37,38].

Our study explores the integration of RPM into public health, analyzing patient-centered care models crucial for health literacy and long-term disease management [10,38]. The real-time data extraction, educational initiatives, and timely follow-up provide insights into RPM development and viability. The findings could contribute to the development of technology-based health management systems in China and abroad to improve hypertension control and management.

## 2. Materials and Methods

This is a multicenter, prospectively designed cohort analysis of 6874 hypertensive patients registered at the Department of General Practice, the second affiliated hospital of Chongqing Medical University, Chongqing, China, and 5 other affiliated hospitals (Daxigou Community Health Service Center, Liberation Monument Community Health Service Center, Qianjiang Central Hospital, Fuling Central Hospital, and Bishan District People’s Hospital). This study was approved by the Institutional Review Board of the Second Affiliated Hospital of Chongqing Medical University (Approval No.: 311/2024). Participants were enrolled prospectively during the RPM phase, whereas pre-RPM clinic data were obtained retrospectively from routine records for the predefined baseline period; analyses were performed after follow-up completion. Written informed consent was obtained from all participants prior to enrollment. Additionally, participants did not receive additional access to antihypertensive drugs or reimbursement of medical expenses beyond what was routinely available as part of standard care.

### 2.1. Inclusion and Exclusion Criteria

Inclusion criteria: Patients aged 18 years or older, both genders (male/female), registered patients in the selected hospitals for this study, with a diagnosis of hypertension irrespective of treatment status, either alone or with at least one comorbidity such as Type 2 diabetes, coronary artery disease, chronic kidney disease, heart failure, ischemic stroke, chronic obstructive pulmonary disease (COPD), or obesity. Participants must have access to mobile phones and agree to use a BP monitoring device, be able to follow the study protocol (such as timely BP recording and data transmission), and be available for follow-up. They must also be willing to provide informed consent.

Exclusion criteria: Newly registered patients, active pregnancy, those using psychiatric drugs, and those with cognitive impairment or end-stage diseases were excluded from the study.

Baseline blood pressure (BP) data from the six months preceding RPM (April 2024 to September 2024) were extracted from hospital records. During the pre-RPM stage, clinic BP measurements were obtained using the HEM-7136 electronic device (National Instrumentation 20262201120; Omron Health Medical Co., Ltd. Dalian, China). Patients rested for 5 min before measurement, remained seated with back supported, avoided talking, and kept the cuff at heart level. BP was measured twice at 1–2 min intervals, and the average was recorded as the visit BP value [39,40]. To characterize routine-care BP capture during the 6-month pre-RPM window, we summarized the frequency and timing of clinic BP measurement visits per patient. Patients had a median of 4 clinic measurement visits (IQR: 2–6), and the median interval between consecutive visits was 45 days (IQR: 28–74 days).

To promote consistency across participating centers for clinic measurements, a standardized protocol was used at all sites, staff followed the same written procedures, and the same device model was used across centers.

Fasting blood glucose (FBG), total cholesterol (TC), and serum creatinine (Scr) were measured at baseline and 6-month follow-up using standard laboratory methods. Antihypertensive and cardiovascular medication use was recorded via electronic health records, including drug classes, dosages, and adjustments (defined as dosage changes, additions, or discontinuations).

Participants received a comprehensive remote blood pressure monitoring intervention for 6 months (October 2024 to March 2025). The study design included two distinct stages: the pre-RPM stage and the post-RPM stage. In the RPM group, each patient was issued an A666G-type (Guangdong Instruments Approval 20172201481) upper-arm electronic BP device manufactured by Ailole medical devices (Shenzhen) Co., Ltd. (Shenzhen, China), which measures BP using the principle of wave measurement and has a built-in SIM card that transmits the patient’s data to the monitoring platform in real time, patients or their family members followed the official WeChat account called “Yikang” with their mobile phone, registered and filled in the personal information, and then scanned the QR code on the device body to bind the device with the mobile phone. After connecting the device, the patient’s blood pressure data were sent to their phone via the official WeChat account message and uploaded to the monitoring platform of the second affiliated hospital of Chongqing Medical University. The patients of the RPM group were asked to measure their BP twice a day. The procedure of measurement was the same as in traditional blood pressure measurement in clinics. During the RPM phase, home blood pressure data were reviewed by clinicians at participating centers through the monitoring platform. When persistently elevated readings were identified, patients were contacted for follow-up, and treatment adjustments were considered based on prevailing hypertension management guidelines and individual clinical circumstances. While no rigid automated treatment algorithm was embedded within the platform, clinical responses followed a structured, guideline-informed approach, allowing clinicians to exercise judgment according to patient age, comorbidities, baseline blood pressure, and treatment tolerance. Additionally, participants at the RPM stage received educational support through the RPM system, including interactive digital content on blood pressure management, medication adherence, lifestyle modifications, and symptom recognition. Personalized feedback messages, reminders, and brief teleconsultations reinforced understanding and self-management. Moreover, the study population consisted of adult and elderly patients. Treatment decisions, including drug selection, were made according to shared clinical guidelines, with the therapeutic target individualized for each patient [40,41,42].

The participation rate was calculated as the proportion of eligible patients who consented. Adherence to RPM was defined a priori as completing ≥80% of the expected home BP measurements during the RPM period. The ≥80% threshold was selected because an 80% cutoff is widely used in adherence research as a pragmatic benchmark to distinguish “high” vs. “lower” adherence, while acknowledging that no single threshold is universally optimal [43,44,45]. Analyses were based on intention-to-treat (ITT), including all enrolled patients regardless of adherence.

**Figure 1:**
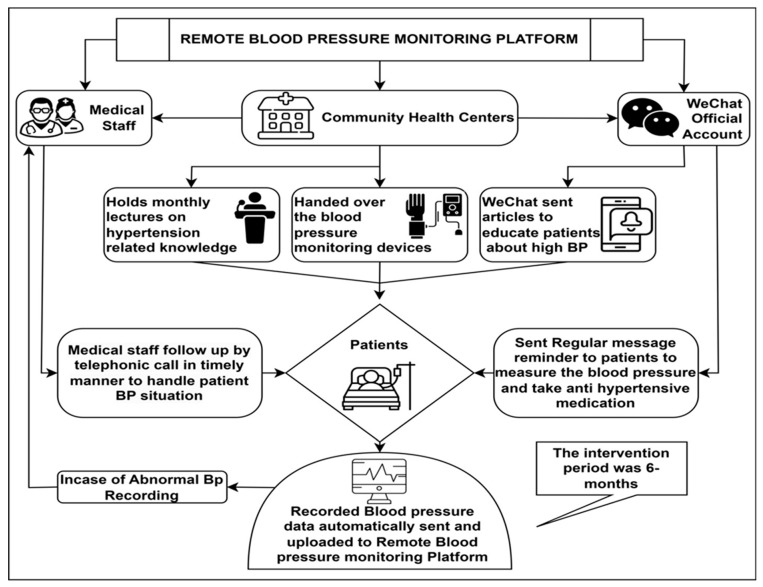
Flowchart of remote patient monitoring.

The specific intervention measures are as follows:

1. The official WeChat account sent reminders to patients to measure their BP and take their antihypertensive medications, along with monthly hypertension-related knowledge [46,47].
2. Patients could remotely monitor their BP through the WeChat application. The monitoring platform allows healthcare providers to view the BP recordings in real time [27], see Figure 1.

### 2.2. Statistical Analysis

Descriptive statistics were computed using SPSS 29.0 (IBM Corporation, Armonk, NY, USA) and Python 3.10 and its scientific libraries (NumPy 1.26, SciPy 1.11, and pandas 2.1) to analyze demographic and clinical data with continuous variables presented as mean ± SD and categorical variables as frequencies and percentages. For each patient, monthly systolic and diastolic blood pressure values were calculated as the arithmetic mean of all home blood pressure readings recorded during that calendar month. No automated outlier exclusion was applied, and all transmitted measurements were included to reflect real-world blood pressure variability during routine RPM use. Measurements were not routinely excluded based on intercurrent illness or medication changes; therefore, monthly averages represent aggregate blood pressure exposure during each study phase rather than stabilized post-titration values. Paired *t*-tests were used to compare within-person changes in systolic and diastolic blood pressure between the pre-RPM and RPM phases. Subgroup analyses stratified by gender, age, baseline blood pressure category, and comorbidity status were performed to explore potential heterogeneity in blood pressure response.

These subgroup analyses were prespecified as exploratory and hypothesis-generating. No formal adjustment for multiple comparisons was applied; therefore, *p*-values from subgroup analyses should be interpreted descriptively rather than as confirmatory evidence. Blood pressure control rates were calculated as proportions meeting predefined thresholds at each time point. Longitudinal trends were summarized using monthly mean blood pressure values over time; however, formal repeated-measures or mixed-effects models were not applied. This approach was chosen to maintain analytical simplicity and transparency, but it does not fully account for within-person correlation across repeated measurements. Data visualization was implemented using Python’s matplotlib and seaborn libraries to compare pre-post measurements, line graphs for BP trajectories, and bar charts illustrating control rates. Statistical significance was set at *p* < 0.05.

## 3. Results

### 3.1. Demographic and Clinical Characteristics

Of 8782 eligible patients, 6874 consented to participate in this longitudinal multicenter study (participation rate 78.3%), with a mean age of 66.9 ± 13.7 years; 48.9% were male. Basic demographic or clinical characteristics of eligible patients who declined participation were not systematically collected, which limits direct assessment of selection bias. Most participants were insured through the employee scheme (62.5%), followed by Urban and Rural Residents Insurance (36.0%), and nearly half were retired (46.2%). The largest regional representation was from Nanan (29.2%), Banan (17.5%), and Yuzhong (13.1%) districts. Cardiovascular disorders were the most common diagnoses, with frequent comorbidities including general symptoms (24.1%) (dizziness, fatigue, weakness), respiratory diseases (18.9%), and cerebrovascular diseases (17.6%) (Table 1). Over six months of RPM-supported care, adherence was high (82.5%, 5678/6874). Paired analyses demonstrated significant improvements across several lifestyle and biochemical parameters. Tobacco and alcohol use declined (*p* < 0.001 and *p* = 0.001, respectively), and significant reductions were observed in body mass index, total cholesterol, fasting blood glucose, and serum creatinine (all *p* < 0.001; Table S1). These changes occurred in parallel with intensified monitoring, clinician follow-up, and medication adjustments during the RPM phase and therefore reflect associations observed during this care period rather than effects attributable solely to RPM technology. Intention-to-treat and per-protocol analyses yielded consistent patterns.

Additionally, the analysis of patient knowledge and management indicators demonstrated profound and statistically significant improvements following the 6-month Remote Patient Management (RPM) intervention (Table S2). Patient awareness and understanding of hypertension, already high at baseline, saw a significant increase. Additionally, crucial metrics related to medication management and overall treatment compliance showed excellent progress, which showed a significant improvement (*p* < 0.001). In short, the implementation of a structured RPM program was associated with significant enhancements across all measured domains of hypertension knowledge, self-management behaviors, and ultimate clinical efficacy, resulting in an improved blood pressure control rate.

### 3.2. Gender-Specific Response Patterns

Gender-stratified analyses revealed differences in observed blood pressure patterns over time. At baseline, mean systolic blood pressure (SBP) was higher among male participants than female participants (142 mmHg vs. 138 mmHg), as was diastolic blood pressure (DBP) (72 mmHg vs. 68 mmHg). During the RPM-supported care period, both groups demonstrated substantial within-person reductions in SBP, with larger early declines observed among males at 1 month (−27 mmHg in males vs. −23 mmHg in females). By 6 months, SBP levels converged, reaching similar values in males (118 mmHg) and females (116 mmHg). Changes in DBP differed modestly between groups, with a greater increase observed among females than males (6 mmHg vs. 4 mmHg) (Table 2). These findings reflect unadjusted, descriptive comparisons and may be influenced by differences in age distribution, comorbidity burden, baseline BP levels, and treatment intensity between genders.

### 3.3. Disease-Specific Responses

Disease-specific subgroup analyses demonstrated variation in observed blood pressure patterns across diagnostic categories (Table 3). All disease groups showed statistically significant within-person reductions in systolic blood pressure (SBP) at 6-month follow-up (all *p* < 0.001), with mean reductions ranging from −22 to −29 mmHg. The largest average SBP declines were observed among participants with musculoskeletal disorders (−29 mmHg; *n* = 245) and cerebrovascular diseases (−28 mmHg; *n* = 333), whereas participants with endocrine and metabolic diseases exhibited smaller mean reductions (−22 mmHg; *n* = 341).

Although between-group differences in SBP change reached statistical significance in unadjusted ANOVA (F [7, 6866] = 4.73, *p* < 0.001), these comparisons should be interpreted cautiously, as disease categories were heterogeneous in composition and size, and analyses were descriptive and unadjusted.

Post-intervention SBP values converged across disease groups (113–116 mmHg), suggesting effective blood pressure normalization during the RPM-supported care period regardless of underlying diagnosis. In contrast, DBP responses were more variable. While most groups showed minimal change (±1–2 mmHg), modest increases were observed among participants with endocrine and metabolic diseases (3 mmHg) and gastrointestinal diseases (2 mmHg), whereas no significant DBP change was detected in several other groups.

Although between-group differences in DBP change were statistically significant, the underlying mechanisms cannot be determined from this observational analysis. The observed DBP patterns may reflect, but do not confirm, interactions among metabolic status, arterial stiffness, autonomic regulation, medication effects, and baseline DBP levels. These interpretations should therefore be regarded as hypothesis-generating rather than causal explanations and warrant further investigation using designs that can account for treatment intensity, vascular function, and time-varying confounding.

The cardiovascular diseases group (*n* = 2007) demonstrated optimal therapeutic response with robust SBP reduction (−23 mmHg) and minimal DBP change (1 mmHg), while respiratory disease patients showed comparable improvements (−24 mmHg SBP) with stable DBP. The convergence of post-intervention SBP values across diverse disease categories represents clinically significant achievement of guideline-recommended targets, though variable DBP responses underscore the need for individualized monitoring, particularly in metabolic conditions.

### 3.4. Blood Pressure Response to RPM Implementation (Overall Blood Pressure Trends)

At baseline, participants had a mean SBP of 140 mmHg and DBP of 72 mmHg. Following initiation of RPM-supported care, SBP declined sharply within the first month (−22 mmHg) and then remained lower through 6 months. DBP showed relative stability, with a slight increase of 1 mmHg from baseline between months 2 and 6, as shown in Table 4.

### 3.5. BP Trajectories

**Figure 2:**
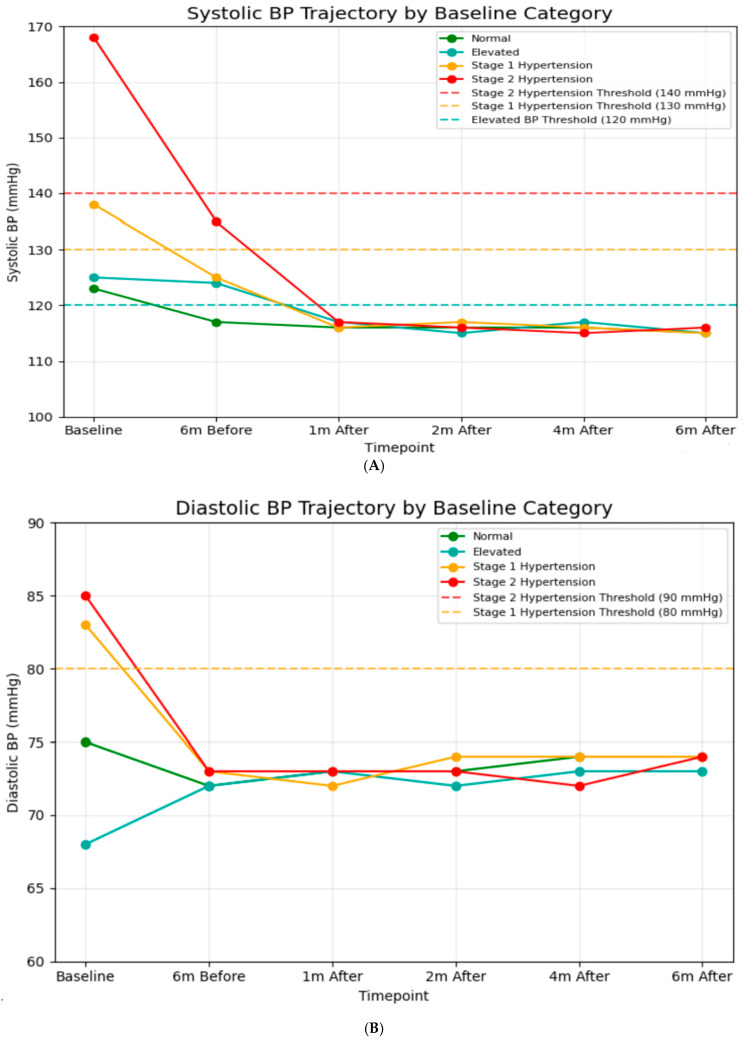
(**A**) Systolic blood pressure trajectory by baseline (*N* = 6874). In baseline: normal; *n* = 2333, elevated; *n* = 298, stage 1 hypertension; *n* = 829, stage 2 hypertension; *n* = 3414. Six months before: normal; *n* = 2789, elevated; *n* = 1416, stage 1 hypertension; *n* = 1320, stage 2 hypertension; *n* = 1349. One month after: normal; *n* = 3762, elevated; *n* = 1267, stage 1 hypertension; *n* = 1227, stage 2 hypertension; *n* = 618. Two months after: normal; *n* = 3963, elevated; *n* = 1292, stage 1 hypertension; *n* = 1278, stage 2 hypertension; *n* = 241. Four months after: normal; *n* = 4041, elevated; *n* = 1312, stage 1 hypertension; *n* = 1248, stage 2 hypertension; *n* = 263. Six months after: normal; *n* = 4056, elevated; *n* = 1291, stage 1 hypertension; *n* = 1280, stage 2 hypertension; *n* = 247. (**B**) Diastolic blood pressure trajectory by baseline (*N* = 6874). In baseline: normal; *n* = 2447, elevated; *n* = 1600, stage 1 hypertension; *n* = 1297, stage 2 hypertension; *n* = 1530. Six months before: normal; *n* = 2396, elevated; *n* = 1654, stage 1 hypertension; *n* = 1504, stage 2 hypertension; *n* = 1319. One month after: normal; *n* = 2504, elevated; *n* = 1543, stage 1 hypertension; *n* = 1498, stage 2 hypertension; *n* = 1329. Two months after: normal; *n* = 2565, elevated; *n* = 1303, stage 1 hypertension; *n* = 1361, stage 2 hypertension; *n* = 1645. Four months after: normal; *n* = 2570, elevated; *n* = 1412, stage 1 hypertension; *n* = 1459, stage 2 hypertension; *n* = 1433. Six months after: normal; *n* = 2549, elevated; *n* = 1409, stage 1 hypertension; *n* = 1451, stage 2 hypertension; *n* = 1465.

A significant difference in baseline SBP was observed among classification groups (Figure 2A). After one month post-intervention, all groups achieved systolic blood pressure readings between 115 and 117 mmHg, which is well below the criterion for hypertension (120 mmHg). This alignment continued during the follow-up period, indicating significant reductions from baseline for hypertensive groups (−52 mmHg for Stage 2), (−22 mmHg for Stage 1). Similarly, baseline DBP values indicated classification categories (Figure 2B). After the intervention, all groups maintained DBP between 71 and 74 mmHg throughout the 6-month follow-up, indicating continuous decreases for hypertensive groups (−11 mmHg for Stage 2, −9 mmHg for Stage 1). Additionally, blood pressure values during the 6-month usual care period were obtained retrospectively from hospital records, and the number and timing of measurements varied among patients. Across the available recordings, mean systolic blood pressure (SBP) was 125 ± 15 mmHg six months prior to the intervention and 140 ± 34 mmHg immediately before the intervention. These findings indicate that some patients already presented with elevated SBP just before entering the digital intervention program.

### 3.6. Blood Pressure Control Rates

**Figure 3:**
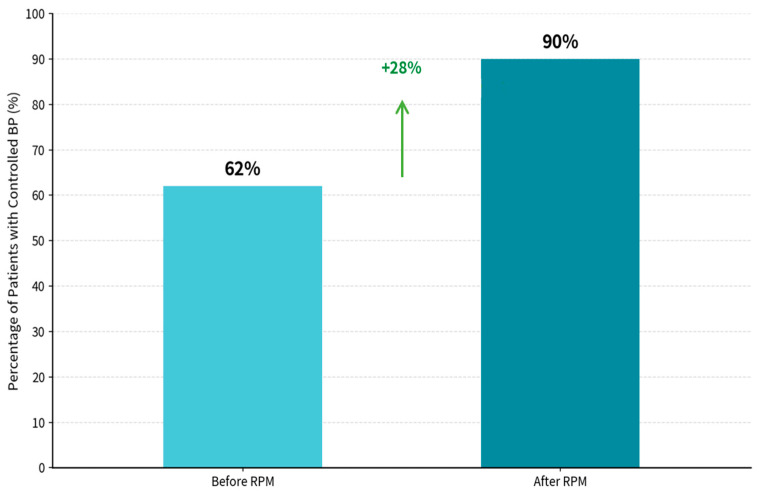
Percentage of patients with controlled blood pressure. BP Control achievement, patients with control BP before RPM: 62.4%, patients with control BP after RPM: 90.1%, and net improvement in controlled BP is 28%. Statistical analysis Results: SBP Paired *t*-test: t = 24.259, *p* < 0.00000000. DBP Paired *t*-test: t = (−2.138, *p* = 0.0208725).

The RPM-supported care period was associated with a marked improvement in BP control, from 62.4% during the pre-RPM phase to 90.1% during the RPM phase (Figure 3). BP control was defined as SBP < 140 mmHg and DBP < 90 mmHg, consistent with commonly used office-based targets in Chinese hypertension guidance and international hypertension guidelines for the general treated population [48,49]. Statistical analysis revealed significant differences between pre- and post-intervention measures for SBP (t = 24.259, *p* < 0.001) and DBP (t = −2.318, *p* = 0.0208725).

### 3.7. Age-Stratified Blood Pressure Trajectories

**Figure 4:**
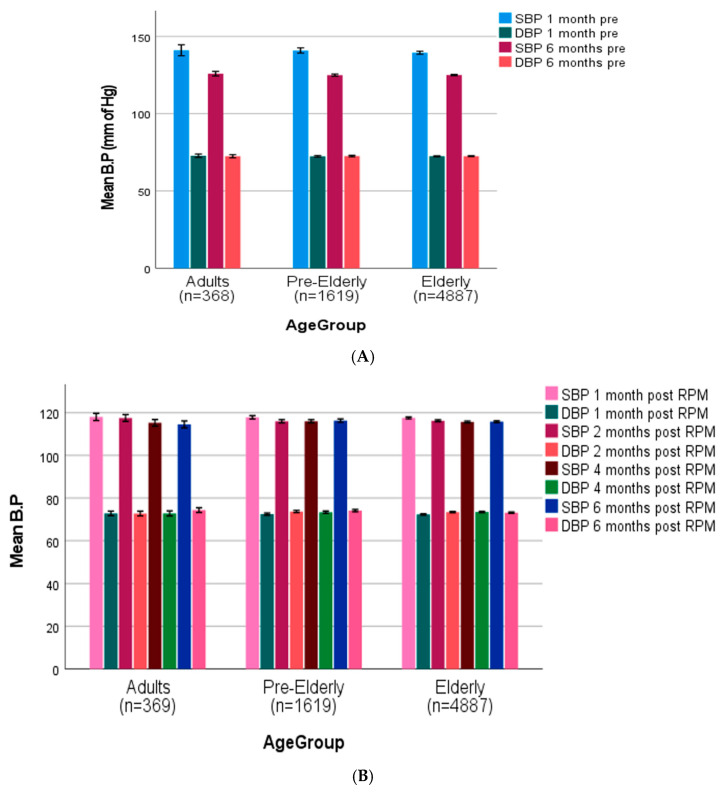
(**A**) Age-stratified blood pressure trajectories before (adult age 20–50 years, pre-elderly 50–64 years, elderly 65 years and above) [50]. (**B**) Age-stratified blood pressure trajectories after remote patient monitoring (adult age 20–50 years, pre-elderly 50–64, elderly 65 and above).

Pre-intervention SBP readings across all age groups (adult age 20–50 years, pre-elderly 50–64 years, elderly 65 years and above) [50] were (adults: 143.7 ± 3.6 mmHg, pre-elderly: 142.5 ± 3.2 mmHg, elderly: 140.8 ± 2.1 mmHg), with slight reductions seen between the 1-month and 6-month pre-intervention intervals (Figure 4A). After RPM intervention, all age groups showed a significant reduction in SBP (119.3 ± 1.9 mmHg, 118 ± 1.7 mmHg, and 118.6 ± 1.5 mmHg in adult, pre-elderly, and elderly groups, respectively) at one-month post-intervention, which remained stable throughout the follow-up time period (Figure 4B). DBP readings showed relative stability during the pre-intervention phase (adults: 72.8 ± 1.7 mmHg, pre-elderly: 72.9 ± 1.5 mmHg, elderly: 72.6 ± 1.3 mmHg) and post-intervention periods in all age groups, with minimal variation seen throughout the study duration. The RPM intervention resulted in significant BP control across all demographic and clinical categories, especially SBP parameters.

### 3.8. Medications

Following initiation of the RPM-supported care period (Table 5), the proportion of participants reporting irregular medication use declined from 11.5% at baseline to 7.5% at 1 month and 2.6% at 6 months (Cochran’s Q = 1958.41, *p* < 0.001). In parallel, treatment intensity increased: monotherapy decreased from 32.6% → 28.9% → 25.5% (Q = 163.40, *p* < 0.001), while two-drug and ≥3-drug regimens increased from 30.6% → 40.1% → 42.0% (Q = 537.99, *p* < 0.001) and 14.3% → 22.6% → 27.9% (Q = 715.12, *p* < 0.001), respectively. By class, use increased for ARBs (28.9% → 35.6% → 37.7%, Q = 318.52, *p* < 0.001), CCBs (31.2% → 42.0% → 43.8%, Q = 781.12, *p* < 0.001), diuretics (13.0% → 22.5% → 24.4%, Q = 684.87, *p* < 0.001), and beta-blockers (22.3% → 24.6% → 25.0%, Q = 31.75, *p* < 0.001), whereas ACEI use was stable (10.0% → 10.5% → 10.7%, Q = 5.71, *p* = 0.058). Together, these patterns suggest that the RPM model primarily functioned as a clinical care enabler, facilitating earlier identification of uncontrolled blood pressure, reinforcing adherence, and supporting clinician-led, guideline-concordant treatment escalation, rather than acting as an isolated therapeutic intervention.

## 4. Discussion

In this pragmatic, multicenter pre–post study, BP control increased from 62.4% to 90.1% during the RPM-supported care period. These findings are directionally consistent with randomized trials and meta-analyses showing that home BP monitoring and telemonitoring, particularly when paired with clinician feedback and medication titration, can improve BP outcomes compared with usual care, although average effect sizes in controlled studies are typically modest [11,51,52].

At the same time, direct quantitative comparison with randomized evidence should be made cautiously because our study lacked a concurrent control group, and the RPM phase coincided with more frequent monitoring, clinician follow-up, and medication intensification, and follow-up BP was measured at home rather than in the clinic. These differences increase susceptibility to confounding, regression to the mean, and measurement-context effects, which may partly explain the larger magnitude of change observed in this real-world evaluation.

The significant reduction in SBP of −22 mmHg within the first month of RPM intervention indicates early effect on blood pressure control is aligned with the research carried out by Boima et al. (2024), which revealed that digital health interventions achieved the most significant reductions in blood pressure within the initial 4–8 weeks of deployment [53]. Our 6-month follow-up showed stable blood pressure. While this pattern is encouraging, the rapid early reduction is also consistent with several alternative explanations that are common in pre–post hypertension evaluations. First, regression to the mean is likely, particularly if enrollment disproportionately included patients with recently elevated clinic readings or uncontrolled BP; repeated measurements over time naturally tend to move toward a patient’s typical range. Second, a change in measurement context may have contributed: baseline values were obtained in the clinic, whereas follow-up readings were collected at home using a connected device, and home BP is often lower than clinic BP due to reduced white-coat effect and more standardized resting conditions. Third, the RPM phase coincided with more active follow-up and treatment adjustment, and our medication data indicates substantial changes in therapy and adherence during this period; these co-interventions plausibly explain a large portion of the early BP reduction. Finally, the pronounced early decline may also reflect behavioral reactivity at the start of monitoring. Increased attention to BP, daily self-measurement, and follow-up contact can transiently improve adherence to medication and lifestyle recommendations (a Hawthorne-type effect), particularly in the first weeks of participation. In addition, enrollment may have preferentially captured patients with recent poor control, which can amplify early apparent improvement in a pre–post design. Taken together, the observed first-month drop should be interpreted as a within-person change during an intensified monitoring and treatment period, rather than an effect attributable solely to RPM technology itself. On the other hand, diastolic blood pressure (DBP) did not show a significant change throughout the study period. Several factors may explain this observation. First, our cohort predominantly presented with systolic hypertension, often associated with preserved diastolic function. Second, the hemodynamic effects of commonly used antihypertensive drugs (e.g., calcium channel blockers) are more pronounced on systolic rather than diastolic pressure. Finally, baseline DBP values were relatively low (72 ± 10 mmHg), leaving limited room for further reduction.

### 4.1. RPM’s Convergence Effect on Blood Pressure Values

A notable observation in this study was the convergence of systolic blood pressure (SBP) values across baseline hypertension categories during the RPM-supported care period. By one month after RPM initiation, participants classified at baseline as normal, elevated, stage 1, or stage 2 hypertension exhibited SBP values within a relatively narrow range (approximately 115–117 mmHg), and this alignment persisted over follow-up.

Importantly, this convergence should not be interpreted as evidence of an intrinsic algorithmic or autonomous control mechanism within the RPM system. Rather, several pragmatic explanations are likely. First, the RPM workflow facilitated protocol-driven treatment titration toward commonly accepted BP targets, supported by frequent home measurements, clinician review, and medication intensification. As shown in Table 5, antihypertensive regimens were progressively escalated, and adherence improved, which would be expected to reduce dispersion in achieved BP values across baseline severity groups. Second, convergence may partly reflect regression to the mean and measurement-context effects, as baseline BP was assessed in clinic settings and follow-up measurements were obtained at home. Patients with higher baseline values would be expected to show larger early reductions under intensified follow-up, while those with lower baseline values may show smaller changes, naturally narrowing between-group differences.

Prior studies have described convergence-like patterns in digital hypertension management, including work exploring personalized or adaptive control frameworks [54,55]. However, such studies typically involve explicit algorithmic feedback or machine-learning–based decision support, which was not implemented in the present program. Therefore, references to theoretical convergence models are best viewed as conceptual parallels rather than mechanistic explanations for the patterns observed here. Taken together, the observed convergence in BP values in this study most plausibly reflects the combined effects of standardized treatment targets, clinician-led titration, improved adherence, and frequent monitoring enabled by RPM, rather than autonomous adaptive control.

### 4.2. Gender-Specific Response Patterns

Our findings of gender-specific variations in blood pressure response to RPM provide significant insights for the modifications of hypertension management. Although both genders reached comparable final systolic blood pressure readings at 6 months (118 mmHg in males, 116 mmHg in females), males showed elevated baseline SBP (142 vs. 138 mmHg) and a more significant absolute drop in SBP at one month (−27 vs. −23 mmHg), but females presented greater absolute increases in DBP (6 vs. 4 mmHg). These findings correlate with the prevailing comprehension of gender disparities in hypertension and response to treatment. Reckelhoff (2001) has thoroughly shown that androgens significantly influence blood pressure control via multiple mechanisms, including the stimulation of the renin-angiotensin system, heightened oxidative stress, and modified pressure-natriuresis connections [56]. The more significant absolute SBP reduction reported in men may indicate that these androgen-mediated mechanisms are more responsive to regular monitoring and treatment modification, and the greater absolute reduction in males can also be explained by a phenomenon known as the ‘baseline effect’, where greater room for improvement often leads to larger observed changes. The reduced baseline in gender disparity in our elderly cohort, compared with younger adults in other studies, aligns with findings from Wahlström M et al., which indicated that post-menopause, women demonstrate increases in blood pressure that progressively approach or exceed those of men, partially attributable to unopposed androgenic effects following the decline in estrogen levels [57]. Remarkably, our RPM intervention seems to neutralize these gender-based disparities, indicating that it may assist in overcoming physiological gaps in treatment response.

### 4.3. Disease-Specific Heterogeneity in Blood Pressure Response

The fluctuations in blood pressure and comorbidity profiles should be considered in hypertension management approaches. The significant reductions in SBP observed in patients with primary hypertension (−40 mmHg) and type 2 diabetes (−40 mmHg) indicate key benefits for these high-risk populations. Furthermore, the fluctuating DBP response across disease categories suggests distinct underlying hemodynamic mechanisms influenced differently by RPM-guided therapy. Patients with diabetes and hypertension often have insulin resistance, sympathetic nerve activation, and other problems, which make blood pressure more difficult to control. RPM plays a role through continuous monitoring, timely intervention, medication management, data-driven methods, etc., thereby making blood pressure control more satisfactory. Mihevc et al. (2024) found that telemonitoring interventions have unique efficacy patterns across various comorbidity profiles, particularly in patients with primary hypertension and cardio-metabolic diseases [58]. The low SBP reductions noted in COPD patients (−14 mmHg) may indicate the complicated relationship between pulmonary pathophysiology and blood pressure regulation, encompassing potential pulmonary vascular remodeling, chronic hypoxemia, and heightened sympathetic activity [59]. These findings emphasize the importance of specialized RPM strategies in particular disease situations.

### 4.4. Age-Related Considerations in RPM Implementation

Our results showed a uniform reduction in SBP across adults, pre-elderly, and elderly groups. This encourages technology use among older people. The SBP reduction across age groups was 119.3 ± 1.9, 118.9 ± 1.7, and 118.6 ± 1.5 mmHg, respectively, at 1 month, suggesting that age does not reduce RPM efficacy. This observation is supported by Parisi (2024), who found that elevated adherence rates to remote monitoring procedures among older participants, indicating that effectively designed digital health treatments may address conventional barriers to technology adoption in this population [60] 10. Furthermore, Li et al. (2023) revealed that remote blood pressure monitoring is “particularly appropriate for those patients where outpatient follow-up is difficult,” a significant issue for elderly individuals with mobility difficulties [4].

### 4.5. Medication Effects

Improved medication regularity emerged as one of the central mechanisms through which RPM enhanced blood pressure control in our cohort. RPM in our cohort was associated with a rapid and sustained intensification of antihypertensive therapy, marked declines in non-adherent status and monotherapy, alongside stepwise increases in dual and triple regimens–patterns that align with contemporary guideline strategy favoring early combination therapy to accelerate blood pressure control. At baseline, 11.5% of patients were non-adherent to prescribed antihypertensive therapy, reflecting real-world gaps in treatment; during RPM, this proportion fell to 2.6%, underscoring its role in improving linkage to and maintenance of pharmacologic therapy. Current guidelines reinforce these observations: the 2023 European Society of Hypertension guideline recommends initiating treatment with two drugs (typically a renin-angiotensin system blocker plus a calcium-channel blocker or thiazide/thiazide-like diuretic), escalating to three agents as needed; the 2017 ACC/AHA guideline similarly advises starting two agents for stage 2 hypertension [41,42]. Consistent with these frameworks, we observed preferential growth in ARB, CCB, and diuretic use, with stable ACE-inhibitor uptake. Mechanistically, RPM likely facilitated timely titration by shortening feedback loops between home BP data and prescribing decisions; prior trials of SMBP/telemonitoring (self-measured blood pressure) show that a meaningful share of BP reduction is mediated by medication adjustment and by team-based, remote communication workflows [61,62]. Finally, our findings concur with recent syntheses indicating that digital health interventions improve BP control across diverse health systems, reinforcing RPM as an effective implementation pathway for guideline-concordant, combination-focused care [51].

### 4.6. Mechanisms Underlying RPM Efficacy

The significance of RPM is regular and systematic blood pressure assessments, which provide more data points than conventional-based monitoring can identify white-coat hypertension, masked hypertension, and blood pressure variability patterns that remain undetected [4]. This improved collected data facilitates more accurate medication adjustments and treatment decision-making. Secondly, RPM likely improves drug adherence by providing regular reminders, facilitating self-monitoring, and encouraging better patient participation. Maglidt (2023) proved that remote monitoring markedly enhanced self-reported adherence to blood pressure medication, an essential element in attaining sustained control [63]. Boima et al. (2024) revealed that digital health interventions enhanced medication adherence in comparison to control factors [53]. Thirdly, RPM may enable timely detection and intervention of blood pressure variations that can prevent target organ damage. The convergence of previously distinct blood pressure categories in our study indicates a “regulatory effect,” whereby RPM assists in stabilizing blood pressure dynamics through timely actions informed by real-time data. Moreover, early SBP reductions (first month) likely reflect the combined effects of medication adjustments and enhanced monitoring. Sustained reductions in subsequent months, however, support RPM’s role in maintaining control through real-time feedback and adherence support.

### 4.7. Strengths, Limitations, and Future Directions

This study has several strengths, including a large multicenter sample, high adherence to RPM, comprehensive subgroup analyses, and the use of intention-to-treat analyses, providing a detailed real-world evaluation of an RPM-supported hypertension management program.

Nevertheless, several important limitations warrant explicit emphasis. First, the absence of a concurrent control group limits causal inference; observed blood pressure changes may reflect regression to the mean, secular trends, or residual confounding rather than effects attributable to RPM alone. The rise in systolic blood pressure immediately prior to enrollment and the rapid early decline after RPM initiation further suggest possible selection effects and behavioral reactivity to intensified monitoring.

Second, blood pressure measurement differed across study phases. Pre-RPM values were obtained from routine clinic records with variable timing and frequency, whereas follow-up measurements during RPM were collected at home using connected devices. This clinic-to-home shift may systematically lower follow-up values and inflate apparent improvements, despite the use of validated devices. Third, medication intensification and improved adherence occurred concurrently with RPM implementation, indicating that pharmacologic optimization is a likely mediator of blood pressure reduction and complicates attribution to RPM technology in isolation.

Additional limitations include limited data on non-participants, potential selection bias related to mobile phone access and digital literacy, reliance on a WeChat-based digital ecosystem, and a relatively short follow-up that precludes assessment of long-term sustainability, cardiovascular outcomes, and cost-effectiveness.

Future studies should employ designs better suited to causal inference while remaining feasible for real-world implementation. In addition to randomized controlled trials, pragmatic cluster-randomized or stepped-wedge designs may be particularly appropriate for evaluating RPM programs embedded within health systems. Longer follow-up, standardized measurement protocols, and formal health-economic evaluations will be essential to assess durability, safety, and scalability across diverse settings and populations.

Importantly, our findings align with the United Nations Sustainable Development Goals (SDGs), particularly SDG 3 (Good Health and Well-being), by promoting health and potentially reducing mortality [64], and SDG 9 (Industry, Innovation, and Infrastructure) [65] by promoting digital/telehealth care, SDG 10 (Reduced Inequalities) [66] through improved accessibility across populations, and SDG 11 (Sustainable Cities and Communities) [67] by fostering community-centered chronic disease management. Finally, RPM has the potential to contribute meaningfully to global agendas for sustainable health systems.

## 5. Conclusions

In this large, multicenter pre–post evaluation, implementation of an RPM-supported hypertension care model was associated with substantial improvements in blood pressure control among patients with comorbidities. Over six months, mean systolic blood pressure declined, and the proportion meeting the study’s control threshold increased, alongside high engagement with home monitoring. During the same period, we observed improved self-management indicators, reduced irregular medication use, and a shift toward more intensive antihypertensive regimens, suggesting that RPM primarily functioned as a practical enabler of closer follow-up and clinician-led treatment optimization in routine care. These findings should be interpreted in light of key limitations, including the absence of a concurrent control group, clinic-to-home measurement differences across phases, and concurrent medication intensification, which together limit causal attribution to RPM technology alone. Future work using pragmatic randomized designs (including cluster or stepped-wedge trials), longer follow-up, standardized outcome measurement, and cost-effectiveness analyses are needed to quantify the independent contribution of RPM and inform scalable implementation.

## Figures and Tables

**Table 1:** Characteristics of the participants who entered the study.

Variables		Values (*N* = 6874)
Gender	Male	3361 (48.9)
Age		66.89 (13.66)
Type of Insurance	Urban and Rural Residents Insurance	2474 (36)
	Insurance for Employees	4298 (62.5)
	Own Expenses	88 (1.3)
	Insurance for Retired People	14 (0.3)
Comorbidity of multiple diseases	General Symptoms	1657 (24.1)
	Cardiovascular Diseases	2007 (29.2)
	Neoplasms	55 (0.8)
	Infectious Diseases	18 (0.3)
	Cerebrovascular Diseases	1210 (17.6)
	Respiratory Diseases	1299 (18.9)
	Neurological Diseases	37 (0.5)
	Gastrointestinal Diseases	265 (3.9)
	Endocrine and Metabolic Diseases	140 (2.0)
	Musculoskeletal Diseases	110 (1.6)
	Circulatory Diseases	52 (0.8)
	Renal Diseases	24(0.3)
Occupation	Self Employed	1108 (16.1)
	Retired Personnel	3179 (46.2)
	Unemployed persons	390 (5.7)
	Farmer	919 (13.4)
	Worker	151 (2.2)
	Staff	962 (14)
	Others	165 (2.4)
Address (districts)	Banan	1204 (17.5)
	Beibei	49 (0.7)
	Jiangjin	68 (1)
	Wanzhou	31 (0.5)
	Dazu	67 (1)
	Jiulongpo	142 (2.1)
	Fuling	95 (1.4)
	Changshou	95 (1.4)
	Rongchang	22 (0.3)
	Tongnan	46 (0.7)
	Sichuan	363 (5.3)
	Nanan	2006 (29.2)
	Chongqing city	499 (7.3)
	Wulong	33 (0.5)
	Yubei	401 (5.8)
	Jiangbei	527(7.5)
	Yuzhong	899 (13.1)
	Shapingba	113 (1.6)
	Liangping	23 (0.3)
	Hechuan	75 (1.1)
	Qianjing	116 (1.7)
Blood Pressure measurements		
SBP 1 month before RPM		140 ± 35
DBP 1 month before RPM		72 ± 10
SBP 6 months before RPM		125 ± 14
DBP 6 months before RPM		72 ± 10
SBP 1 month after RPM		118 ± 16
DBP 1 month after RPM		72 ± 10
SBP 2 months after RPM		116 ± 15
DBP 2 months after RPM		73 ± 11
SBP 4 months after RPM		116 ± 15
DBP 4 months after RPM		73 ± 11
SBP 6 months after RPM		116 ± 15
DBP 6 months after RPM		73 ± 11

Note: Continuous data are shown as mean ± SD, and categorical data are represented as frequencies (percentages). Systolic blood pressure (SBP), diastolic blood pressure (DBP), and general symptoms (dizziness, fatigue, weakness).

**Table 2:** Gender differences and time frame trends in BP changes.

Time Point	Gender	Mean SBP	Change in SBP from 1 Month Before	Mean DBP	Change in DBP from 1 Month Before
1 month Before	Male	140 ± 34	0	72 ± 10	0
1 month Before	Female	140 ± 35	0	72 ± 10	0
6 Months Before	Male	125 ± 15	−15	72 ± 10	+0
6 Months Before	Female	125 ± 15	−15	73 ± 10	+1
1 Month After	Male	118 ± 16	−22	72 ± 10	+0
1 Month After	Female	117 ± 16	−23	72 ± 10	+0
2 Months After	Male	116 ± 15	−24	74 ± 11	+2
2 Months After	Female	116 ± 16	−24	73 ± 11	+1
4 Months After	Male	116 ± 15	−24	74 ± 11	+2
4 Months After	Female	116 ± 15	−24	73 ± 11	+1
6 Months After	Male	116 ± 15	−24	74 ± 11	+2
6 Months After	Female	116 ± 15	−24	73 ± 11	+1

Data are shown as a mean ± SD, blood pressure measured in mm of Hg. Negative sign shows a decrease in BP, while a positive sign shows an increase in BP (male *n* = 3361; female *n*= 3513).

**Table 3:** Blood pressure in different disease groups emphasizes disease-specific variability and the six-month follow-up period.

Diagnosis Group (*N* = 6874)	1 Month Before SBP (Baseline)	1 Month Before DBP (Baseline)	6 Months After SBP	6 Months After DBP	SBP Change	*p*-Value (Within Group)	DBP Changes	*p*-Value (Within Group)
Cardiovascular diseases (*n* = 2007)	139 ± 35	72 ± 10	116 ± 15	73 ± 11	−23	<0.001	1	<0.001
Endocrine and metabolic diseases (*n* = 341)	138 ± 34	73 ± 10	116 ± 15	76 ± 11	−22	<0.001	3	<0.001
Respiratory diseases (*n* = 1298)	140 ± 34	73 ± 10	116 ± 15	74 ± 11	− 24	<0.001	1	<0.001
Cerebrovascular diseases (*n* = 333)	141 ± 35	72 ± 11	113 ± 15	73 ± 11	−28	<0.001	1	0.130
Gastrointestinal diseases (*n* = 600)	140 ± 35	72 ± 10	115 ± 15	74 ± 11	−25	<0.001	2	<0.001
Musculoskeletal disorders (*n* = 245)	144 ± 34	73 ± 10	115 ± 16	74 ± 11	−29	<0.001	1	0.069
General (*n* = 1654)	140 ± 35	72 ± 10	116 ± 15	73 ± 11	−24	<0.001	1	<0.001
Others (*n* = 396)	139 ± 31	75 ± 12	114 ± 15	74 ± 11	−25	<0.001	−1	0.137
Analysis of Variance (Between-Group Comparison of Change Scores)								
Measure		Statistical Test		Result		*p*-value (Between Group)		
SBP Change (ΔSBP)		One-Way ANOVA		F (7, 6866) = 4.73		<0.001		
DBP Change (ΔDBP)		One-Way ANOVA		F (7, 6866) = 7.85		<0.001		

Data are shown as mean ± SD; average blood pressure (mm of Hg). Negative signs show a decrease in BP, while positive signs show an increase in BP. SBP (systolic blood pressure), DBP (diastolic blood pressure), general (dizziness, fatigue, weakness), others (neoplasm, infectious diseases, neurological diseases, renal diseases). 1. Within-group analysis: A paired-samples *t*-test was conducted for each disease group to compare pre-RPM and post-RPM blood pressure. All *p*-values are two-sided. 2. Between-group analysis: A one-way ANOVA was conducted to determine if the magnitude of blood pressure change (Δ) differed across the eight diagnosis groups. The ANOVA results indicate a statistically significant difference between groups for both ΔSBP and ΔDBP. 3. Post hoc testing: Due to the significant ANOVA results, post hoc tests (Tukey HSD) are required to identify which specific disease groups differ from each other in their response to RPM. For example, the musculoskeletal disorders group (ΔSBP = −29) and the cerebrovascular diseases group (ΔSBP = −28) showed numerically greater SBP reductions, while the endocrine and metabolic diseases group showed a greater DBP increase (ΔDBP = +3). The post hoc analysis will clarify if these numerical differences are statistically significant.

**Table 4:** Blood Pressure Changes Before and After Remote Patient Management (RPM) Intervention.

Time Point	SBP (mmHg)	DBP (mmHg)	*p*-Value vs. Baseline (SBP)	*p*-Value vs. Baseline (DBP)
1 month before (baseline)	140 ± 35	72 ± 10	—	—
1 month after	118 ± 16	72 ± 10	<0.001	>0.999
2 months after	116 ± 15	73 ± 11	<0.001	0.157
4 months after	116 ± 15	73 ± 11	<0.001	0.157
6 months after	116 ± 15	73 ± 11	<0.001	0.157

Note: Data are presented as “mean ± SD.” *p*-values were calculated using a paired *t*-test, comparing each time point to the baseline value.

**Table 5:** Antihypertensive medication use at baseline and during follow-up.

Medication Class/Number of Agents	Baseline (Pre-RPM) *n* (%)	1 Month Post-RPM *n* (%)	6 Months Post-RPM *n* (%)	Cochran’s Q Value	*p*	Odds Ratio (95% CI) for 6 Months vs. Baseline	Effect Size (Cramér’s V)
Irregular medication	790 (11.5)	517 (7.5)	181 (2.6)	1958.4	<0.001	0.21 (0.18–0.25)	0.38
ACE Inhibitor	687 (10.0)	721 (10.5)	735 (10.7)	5.7	0.058	1.07 (0.96–1.19)	0.02
ARB	1988 (28.9)	2450 (35.6)	2589 (37.7)	318.5	<0.001	1.50 (1.40–1.61)	0.15
Beta-Blocker	1531 (22.3)	1689 (24.6)	1722 (25.0)	31.7	<0.001	1.16 (1.08–1.25)	0.05
CCBs	2145 (31.2)	2890 (42.0)	3011 (43.8)	781.1	<0.001	1.72 (1.61–1.84)	0.21
Diuretic	895 (13.0)	1550 (22.5)	1680 (24.4)	684.9	<0.001	2.16 (1.98–2.36)	0.23
Number of Agents							
0	790 (11.5)	517 (7.5)	181 (2.6)	1958.4	<0.001	0.21 (0.18–0.25)	0.38
1	2240 (32.6)	1989 (28.9)	1754 (25.5)	163.4	<0.001	0.71 (0.66–0.76)	0.09
2	2101 (30.6)	2756 (40.1)	2890 (42.0)	537.9	<0.001	1.64 (1.53–1.76)	0.16
≥3	986 (14.3)	1552 (22.6)	1918 (27.9)	715.1	<0.001	2.34 (2.14–2.56)	0.22

Notes: Cochran’s Q test was used to compare the differences in medication usage across the three time points (baseline, 1-month, and 6-months) for the same cohort (*N* = 6874). A significant Cochran’s Q test (*p* < 0.05) indicates that the proportion of patients changed significantly over time. Post hoc pairwise comparisons with Bonferroni correction (significance level set at *p* < 0.01 for each pair) were performed for all significant outcomes. All significant overall differences were driven by significant changes between baseline vs. 1-month and baseline vs. 6-month (*p* < 0.001 for all relevant comparisons). The change from 1 month to 6 months was also significant for most outcomes, but of smaller magnitude. The exception is ACE inhibitor use, where the change across the three time points was not statistically significant (Q = 5.71, *p* = 0.058). CCB (calcium channel blocker), ACE (angiotensin-converting enzyme), ARB (angiotensin receptor blocker). Irregular medication: intermittent medication, irregular medication frequency/time/dosage, incorrect medication method.

## Data Availability

The data presented in this study are available on request from the corresponding author due to privacy restrictions.

## Supplementary Materials

The following supporting information can be downloaded at: https://www.mdpi.com/article/10.3390/diagnostics16020244/s1, Table S1: Comparison of Clinical Characteristics Before and 6 Months After Remote Patient Management (RPM); Table S2: Comparison of Hypertension Knowledge and Management Indicators Before and 6 Months After RPM.
