# Supplementary material for: Association of Mobile-Enhanced Remote Patient Monitoring with Blood Pressure Control in Hypertensive Patients with Comorbidities: A Multicenter Pre–Post Evaluation
Source: Diagnostics (Basel). 2026 Jan 12;16(2):244. doi: 10.3390/diagnostics16020244 (PMC12840447; doi:10.3390/diagnostics16020244)
Supplement: Supplementary file 1 [file diagnostics-16-00244-s001.zip › diagnostics-4038704-supplementary.pdf]

Table S1. Comparison of Clinical Characteristics Before and 6 Months After Remote Patient Management (RPM)

Indicator	Pre-RPM (N=6874)	6 Months Post-RPM (N=6874)	Statistical Value	p value
Tobacco uses history, n (%)	3022 (43.96)	2740 (39.85)	$\chi^2 = 43.56$[1]	<0.001
Alcohol uses history, n (%)	1686 (24.53)	1556 (22.64)	$\chi^2 = 10.88$[1]	0.001
Average 24-hr pulse pressure, mm Hg	73.92 ± 11.32	65.79 ± 9.11	t = 45.92[2]	<0.001
Average 24-hr HR, beat/min	78.17 ± 15.20	70.58 ± 6.49	t = 36.52[2]	<0.001
BMI, kg/m²	24.25 ± 0.69	23.95 ± 0.62	t = 28.57[2]	<0.001
Total Cholesterol (TC), mmol/L	5.34 ± 1.12	5.02 ± 1.03	t = 19.78[2]	<0.001
Serum creatinine (Scr), µmol/L	91.17 ± 12.13	88.02 ± 7.67	t = 20.30[2]	<0.001
Fasting Blood Glucose (FBG), mmol/L	6.23 ± 1.54	5.81 ± 1.32	t = 18.33[2]	<0.001

Notes: Data are presented as Mean ± Standard Deviation (SD) or number (n) and percentage (%). [1] χ^2 value represents the test statistic, [2] t value represents the paired-samples t-test statistic. All *P* values are derived from comparisons between the Pre-RPM and 6 Months Post-RPM groups using the appropriate paired tests. $p < 0.05$ was considered statistically significant.

Table S2. Comparison of Hypertension Knowledge and Management Indicators Before and 6 Months After RPM

Indicator	Pre-RPM (N=6874)	6 Months Post-RPM (N=6874)	χ^2 (McNemar)	*P*
Awareness rate of hypertension	91.25% (6272)	98.57% (6776)	254.71	<0.001
Medication adherence rate	86.42% (5940)	95.42% (6559)	294.73	<0.001
Treatment compliance rate	86.19% (5925)	93.48% (6425)	222.60	<0.001
Regular exercise rate	78.69% (5409)	84.26% (5791)	87.29	<0.001
Diet improvement rate	75.39% (5182)	85.42% (5871)	233.27	<0.001

Note: Data is represented as percentage and frequencies. χ^2 value represents the chi-square value (df=1). $p < 0.05$ was considered statistically significant.
